# Dynamic motion analysis of the cervical spine during normal swallowing

**DOI:** 10.1097/MD.0000000000044168

**Published:** 2025-09-05

**Authors:** Kojiro Mekata, Hotaka Takizawa, Tomoyuki Takigawa, Yasuo Ito

**Affiliations:** a Department of Rehabilitation, Shijonawate Gakuen University, Osaka, Japan; b Department of Computer Science, Graduate School of Systems and Information Engineering, University of Tsukuba, Ibaraki, Japan; c Department of Orthopaedic Surgery, Kobe Red Cross Hospital, Hyogo, Japan.

**Keywords:** biomechanical phenomena, cervical vertebrae, computer-assisted, deglutition, fluoroscopy, radiographic image interpretation, spine

## Abstract

This study aims to clarify the dynamic changes in the cervical lordotic angle (CLA) during normal swallowing using an automated motion analysis method. Physiological cervical lordosis is crucial for spinal alignment and musculoskeletal function. While previous studies have noted the relevance of cervical curvature in clinical contexts, its dynamic modulation during swallowing has not been well studied. Videofluoroscopic swallowing examinations were conducted in 39 healthy individuals without cervical spine disease. A 2-dimensional template matching technique was applied to automatically track the cervical spinous processes and vertebral bodies over 61 frames spanning 1 second before and after the pharyngeal phase. The CLA was calculated using the angle between the cervical baseline (fitted to vertebral centroids) and reference lines (drawn between vertebral body and spinous process). Subjects were categorized by baseline lordotic angle into 4 groups: <0°, 0° to 10°, 10° to 20°, and ≥20°. Across all participants, the CLA decreased during the pharyngeal phase, peaking at the time of maximum hyoid elevation. Group-wise analysis revealed minimal change in the < 0° group. The 0° to 10° group showed early reduction before the pharyngeal phase. The 10° to 20° group demonstrated the most synchronized and prominent angular transition centered around the pharyngeal phase. In the ≥ 20° group, lordosis decreased but showed limited restoration post-swallowing. Cervical spine motion during swallowing varies with baseline lordotic angle. Moderate lordosis (10–20°) is associated with the most efficient and coordinated motion. This suggests that optimal cervical alignment may facilitate safe and effective deglutition. Quantifying such dynamic motion could support future diagnostic tools and rehabilitation strategies for dysphagia or cervical dysfunction.

## 1. Introduction

Extensive research has been conducted on the dynamics of human bone movement, particularly in relation to gait, posture, and rehabilitation. However, the specific movements of the cervical spine during swallowing have not yet been fully elucidated. Swallowing is a complex biomechanical process involving coordinated activity between oropharyngeal structures and the cervical spine. Understanding cervical spine kinematics during this process is essential, as cervical alignment may affect swallowing efficiency and safety, especially in clinical populations with dysphagia or musculoskeletal disorders.

The physiological lordosis of the cervical spine plays an important role in maintaining head posture and spinal stability. Prior studies have shown that cervical lordotic angles (CLAs) vary with age and sex,^[1]^ and have suggested associations with neck pain.^[2]^ Therapeutic interventions such as cervical traction can temporarily increase cervical lordosis and alleviate pain.^[3]^ Furthermore, loss of the cervical lordotic curvature has been linked to weakening of the cervical extensor muscles, potentially contributing to postural dysfunction.^[4]^ Despite these findings, the behavior of the CLA during swallowing remains poorly understood.

In our previous study, we conducted videofluoroscopic swallowing examinations (VF) on 39 healthy individuals and analyzed cervical motion using ImageJ software (National Institutes of Health, Bethesda) at 2 characteristic time points: the oral and pharyngeal phases of swallowing. The results revealed a transient reduction in the CLA during the pharyngeal phase.^[5]^ However, that analysis method had key limitations: it could not be applied when spinous processes were altered by surgery, and cervical structures had to be manually segmented in each video frame, making large-scale analysis and dynamic tracking difficult.

To overcome these limitations, we developed an engineering-based approach using 2-dimensional template matching to automatically track cervical spinous processes and vertebral bodies in VF videos.^[6–8]^ While the initial template region must be set manually in the first frame, subsequent frames can be tracked automatically, allowing for time-efficient and objective motion analysis across multiple frames.

In the present study, we applied this semi-automated method to extract the coordinates of the spinous processes and vertebral bodies over 61 consecutive frames, spanning 1 second before and after the pharyngeal phase. From these data, we calculated the CLA using the centroids of each tracked structure. This study aims to clarify dynamic changes in the CLA during normal swallowing and to characterize patterns of reduction and restoration based on interindividual differences in baseline cervical lordosis. Our findings may help define the optimal cervical alignment for effective swallowing and contribute to the development of new assessment methods and rehabilitation strategies for individuals with dysphagia or cervical spine dysfunction.

## 2. Methods

### 2.1. Participants

This study included healthy adult volunteers with no history of cervical spine or neck-related disorders. The inclusion criteria were: adult age as generally defined for research participation; and absence of any history of cervical or neck disease. The exclusion criteria were: prior cervical spine surgery; presence of neurological disorders; and history of recent radiation exposure.

A total of 39 healthy individuals (23 males and 16 females; mean age 34.3 ± 8.2 years, mean ± SD) participated in the study. All participants received a thorough explanation of the study and provided written informed consent prior to participation. Participation was voluntary, and subjects were free to withdraw at any time without consequence. The study was conducted in accordance with the ethical standards of the institutional ethics committee.

### 2.2. VF study

The VF study was conducted in a manner similar to that described in a previous study^[5]^ and based on the manual^[9]^ created by The Japanese Society of Dysphagia Rehabilitation. The X-ray fluoroscopy device used was CUREVISTA (Hitachi Medico, Tokyo, Japan), and the video recording device was DMCAT-2000HL (Panasonic, Osaka, Japan). The filming direction was lateral, and the images were recorded at 30 fps. The subjects sat on a chair with their trunk inclined and neck at a 90° angle to the ground and self-administered the contrast agent using a paper cup. The contrast agent was 40% diluted barium sulfate solution (10 mL). A 1 cm scale was filmed at the start of the recording.

### 2.3. Image analysis

**Figure 1.**
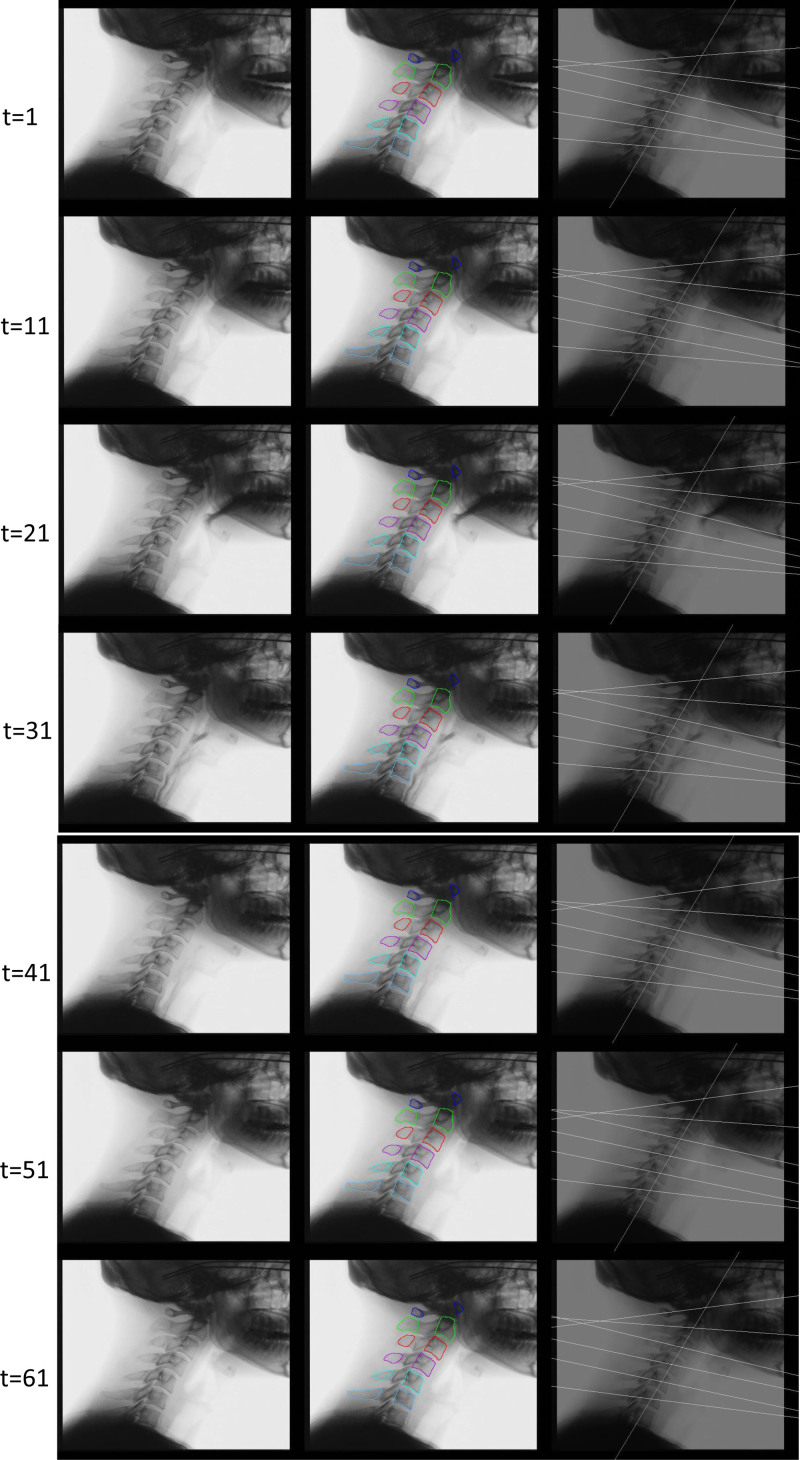
VF video sample of a healthy volunteer. The right and center of the figure show the original image and tracking image obtained by template matching, respectively. The left side shows the results obtained using the cervical baseline and cervical reference lines used to determine the cervical lordotic angle. The pharyngeal phase is indicated by t = 31.

Templates of each cervical vertebra (vertebral body and spinous process) were created from the videofluoroscopic images, and an engineering method, 2-dimensional template matching, was utilized to develop a method to automatically track the cervical spine (cervical spinous processes and vertebral bodies) in VF videos.^[6–8]^ This method requires manual setting of the template surrounding the tracking target areas (cervical spinous processes and vertebral bodies) in the first tracking frame; however, subsequent frames can be tracked automatically (Fig. 1B). In this study, the cervical spinous processes and vertebral bodies were extracted over 61 frames spanning 1 second before and after the pharyngeal phase, and the centroids of each cervical vertebral body and spinous process in each frame image were determined to calculate the angles of each cervical vertebra. An example of these results is shown in Figure 1C (Supplemental Video File 1). The pharyngeal phase is defined as the frame with maximum anterior–superior elevation of the hyoid bone, and the oral phase is defined as 30 frames before the pharyngeal phase.

**Figure 2.**
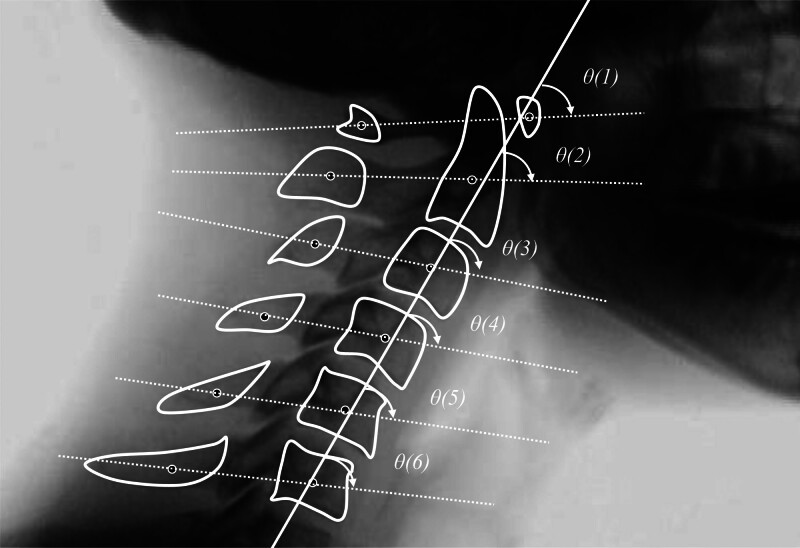
Defines the line that connects the centroids of body of each cervical vertebra and spinous process as the cervical reference line (white dotted line). The line fitted to the centroids of each cervical vertebral body is defined using the least-squares method as the cervical baseline (white solid line). The angle formed between the cervical baseline and each cervical reference line is defined as θ.

As shown in Figure 2, the angles of each cervical vertebra are determined by fitting a straight line to the centroids of the vertebral bodies in each frame using the least-squares method. This line is defined as the cervical baseline (solid line in Fig. 2). Additionally, a straight line that connects the centroids of the vertebral body and spinous process of each cervical vertebra is defined as the cervical reference line (dotted line in Fig. 2). The angle between the cervical baseline and reference line of each cervical vertebra was measured. In this study, the first and second cervical vertebrae are defined as the upper cervical vertebrae, and the third and below as the lower cervical vertebrae. The difference between the average angles of the upper and lower cervical vertebrae is defined as the CLA.

The CLA of each subject was determined during the oral phase and defined as the physiological lordotic angle. These angles were defined and measured at second to sixth cervical vertebrae (C2–C6) and classified into 4 categories for comparison: a = <0°; b = ≥0° but < 10°; c = ≥10° but < 20°; and d = ≥20°.

## 3. Results

**Figure 3.**
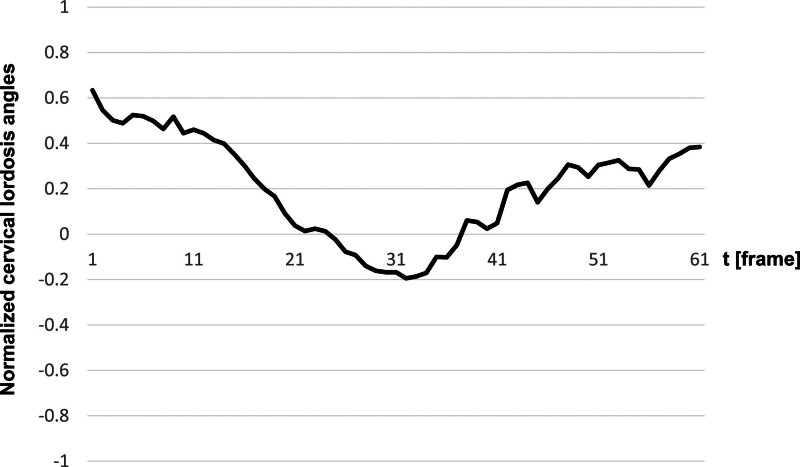
The average transition of lordotic angles. The horizontal and vertical axes represent time and normalized lordotic angle, respectively.

Of the 39 participants, the results of 38 were analyzed, excluding one for whom image processing was difficult, as shown in Figure 3. In this normal cohort, the CLA decreased during the pharyngeal phase (t = 31) compared to that during the oral phase (t = 1), with a peak reduction occurring during the pharyngeal phase. Evidently, nearly full restoration occurred 1 second after the pharyngeal phase (t = 61). Additionally, the temporal motion analysis suggests that swallowing and cervical spine movements were synchronized and concluded in < 2 seconds.

**Figure 4.**
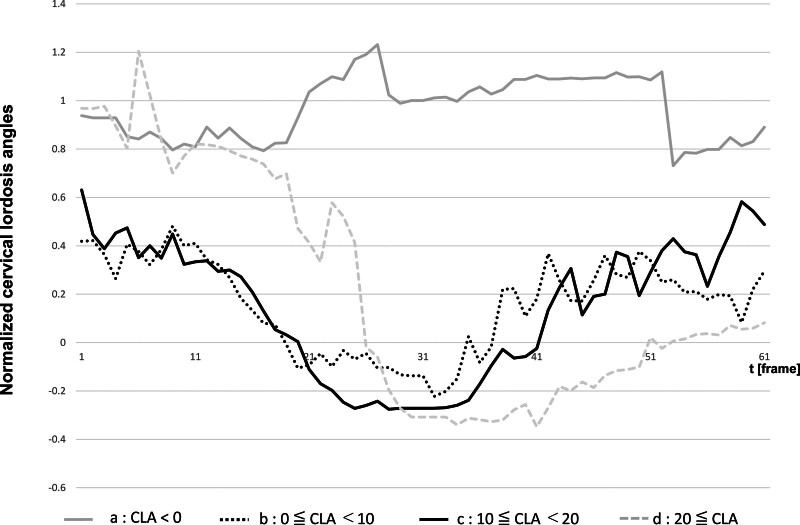
(A) CLA < 0, (B) 0 ≤ CLA < 10, (C) 10 ≤ CLA < 20, and (D) CLA ≥ 20. CLA = cervical lordotic angle (°).

The transition of the physiological CLAs for each participant is shown in Figure 4. The distribution is 2 (5.3%), 12 (31.6%), 18 (47.4%), and 6 (15.8%) for categories a to d, respectively. For the average transition of lordotic angles, category a showed almost no movement. Category b peaked around the pharyngeal phase, but an overall decrease occurred before the pharyngeal phase, approximately between frames 20 and 30. Category c shows a peak decrease around the pharyngeal phase within 10 frames before and after. Category d experiences a decrease around the pharyngeal phase, but restoration is not observed even 1 second after the pharyngeal phase.

## 4. Discussion

The results indicate that the CLA decreased during the pharyngeal phase (t = 31) compared to that during the oral phase (t = 1). This finding is consistent with our previous findings^[5]^; however, in this study, temporal changes were observed over 2 seconds before and after the pharyngeal phase, which was not reported previously, making it a valuable contribution to the study of cervical spine dynamics during swallowing. Specifically, nearly the same transition as in the oral phase was confirmed to return 1 second after the pharyngeal phase (t = 61), and a rapid decrease and recovery were observed immediately after the oral and pharyngeal phases, respectively. This is consistent with reports that swallowing movements occur within 2 seconds.^[10]^ During normal swallowing, nasopharyngeal closure, maximum anterior–superior elevation of the hyoid bone, and laryngeal elevation occur almost simultaneously, similar to the opening of the esophageal entrance and laryngeal closure. At the time of maximum anterior–superior elevation of the hyoid bone (pharyngeal phase), the throat and neck muscles act in synchrony.^[11]^ The temporal analysis results of this study support the idea that the peak of CLA reduction coincides with the pharyngeal phase in the normal group. In other words, swallowing and cervical spine movements are synchronized during normal swallowing. These results are expected to serve as fundamental data for comparative studies in groups with swallowing disorders or cervical spine abnormalities, and identifying changes in the CLA in abnormal groups requires imaging for longer than 2 seconds centered around the pharyngeal phase, especially more than 1 second after the pharyngeal phase. The problems with the number of cases and the reduction of radiation exposure for imaging time make it difficult to immediately classify the entire image of the CLA in groups with cervical abnormalities. Thus, elucidating the cervical spine and swallowing movements in these groups is considered a crucial issue for future research.

Furthermore, the transition of the physiological CLAs in each participant suggest its probable involvement in swallowing movements. That is, a relatively small physiological lordotic angle may result in contraction of muscles related to swallowing, occurring and concluding before the pharyngeal phase. Similarly, a relatively large physiological lordotic angle may require the muscles involved in swallowing to continue contracting even after the pharyngeal phase. This suggests that an angle of physiological lordosis can make swallowing difficult. Research on physiological lordosis of the cervical spine has been reported, including measurement methods starting with X-rays,^[12,13]^ their standardization,^[13–15]^ and their association with diseases.^[16–18]^ However, no studies have been conducted on dynamic transitions. The results of this study provide basic data on the effects of the physiological lordotic angle of the cervical spine on the dynamics during swallowing. Notably, most cases in this study followed patterns b (12/38 participants (31.6%)) and c (18/38 participants (47.4%)), and patterns a (2/38 participants (5.3%)) and d (6/38 participants (15.8%)) were slightly rare. Thus, it is necessary to increase the number of cases in the future to examine the distribution of physiological lordotic angles in the normal group.

In addition, while the physiological lordotic angle is commonly calculated from C2 to C7, tracking C7 is difficult owing to image processing problems; therefore, in this study, C2 to C6 was defined as the physiological lordotic angle. In the future, improving the accuracy of the image processing technology and conducting comparisons using the commonly used C2 to C7 will be necessary.

The criteria for diagnosis and evaluation using VF are based on the characteristics of radiographs; however, the results of this study suggest that new diagnostic and evaluation criteria may be required for the assessment of cervical abnormalities and swallowing movements. This suggests that joint movement of the cervical spine is necessary for normal swallowing, and natural swallowing may be hindered if the joint movement of the cervical spine is restricted. Clarifying the extent to which the cervical spine movement affects swallowing is an important topic for future research.

The results of this study can be used as basic data on cervical spine movement during swallowing. However, in addition to the number of cases mentioned earlier, the study has several limitations. For example, this study mainly targeted data from younger individuals; data from older adults were not included. Additionally, this study conducted swallowing evaluations using liquids in a seated position for the analysis of normal swallowing. Evaluations using a supine position or foods with higher viscosity were not conducted, leaving these as future research topics.

This study clarified that physiological lordosis of the cervical spine decreases during swallowing; however, further research is needed to understand the functional significance of this finding.

## Author contributions

**Conceptualization:** Kojiro Mekata.

**Data curation:** Kojiro Mekata.

**Formal analysis:** Kojiro Mekata.

**Investigation:** Kojiro Mekata.

**Methodology:** Kojiro Mekata.

**Project administration:** Kojiro Mekata.

**Resources:** Kojiro Mekata.

**Software:** Kojiro Mekata.

**Supervision:** Kojiro Mekata, Tomoyuki Takigawa, Hotaka Takizawa, Yasuo Ito.

**Validation:** Kojiro Mekata.

**Visualization:** Kojiro Mekata.

**Writing – original draft:** Kojiro Mekata.

**Writing – review & editing:** Kojiro Mekata.
